# Homotaurine limits the spreading of T cell autoreactivity within the CNS and ameliorates disease in a model of multiple sclerosis

**DOI:** 10.1038/s41598-021-84751-3

**Published:** 2021-03-08

**Authors:** Jide Tian, Min Song, Daniel L. Kaufman

**Affiliations:** Department of Molecular and Medical Pharmacology, David Geffen School of Medicine, UCLA School of Medicine, University of California, Los Angeles, Los Angeles, CA 90095-1735 USA

**Keywords:** Autoimmunity, Immunotherapy

## Abstract

Most multiple sclerosis (MS) patients given currently available disease-modifying drugs (DMDs) experience progressive disability. Accordingly, there is a need for new treatments that can limit the generation of new waves T cell autoreactivity that drive disease progression. Notably, immune cells express GABA_A_-receptors (GABA_A_-Rs) whose activation has anti-inflammatory effects such that GABA administration can ameliorate disease in models of type 1 diabetes, rheumatoid arthritis, and COVID-19. Here, we show that oral GABA, which cannot cross the blood–brain barrier (BBB), does not affect the course of murine experimental autoimmune encephalomyelitis (EAE). In contrast, oral administration of the BBB-permeable GABA_A_-R-specific agonist homotaurine ameliorates monophasic EAE, as well as advanced-stage relapsing–remitting EAE (RR-EAE). Homotaurine treatment beginning after the first peak of paralysis reduced the spreading of Th17 and Th1 responses from the priming immunogen to a new myelin T cell epitope within the CNS. Antigen-presenting cells (APC) isolated from homotaurine-treated mice displayed an attenuated ability to promote autoantigen-specific T cell proliferation. The ability of homotaurine treatment to limit epitope spreading within the CNS, along with its safety record, makes it an excellent candidate to help treat MS and other inflammatory disorders of the CNS.

## Introduction

Despite the availability of DMDs with different modes of action, most MS patients experience disease relapses and progressive disability. Accordingly, there is a need for novel treatments that can more effectively limit the waves of T cell autoreactivities to components of myelin that are thought to drive disease relapses. Several lines of evidence suggest that the activation of γ-aminobutyric acid receptors (GABA-Rs) may provide a new approach to safely help regulate pathogenic autoimmune responses in MS.

GABA is well known as the major inhibitory neurotransmitter in the adult CNS. Interestingly, many different types of murine and human immune cells also express GABA-Rs. Several groups have shown that GABA limits murine T cell production of IL-2^1^, IFNγ^2–4^, TNFα^3^, and IL-12^3^ while promoting TGFß and Treg responses^3–5^. APCs such as macrophages, dendritic cells, and microglia also express GABA_A_-Rs and their activation inhibits their inflammatory activity^6–11^. For examples, GABA or a GABA_A_-R agonist reduced the secretion of IL-6, IL-1ß, IL-12 and/or TNFα from LPS-stimulated murine macrophages or dendritic cells^7,11,12^. Taking advantage of these anti-inflammatory effects, several different research groups have shown that GABA administration ameliorated disease in mouse models of T1D, RA, and T2D^1–3,5,6,13–15^. Moreover, we recently showed that GABA treatment effectively prevented severe illness and death in coronavirus-infected mice when this treatment was initiated after the appearance of symptoms^16^.

Human T cells also express GABA_A_-Rs that can be modulated by GABA_A_-R agonists and antagonists^13,14,17,18^. GABA inhibited human PBMC proliferative responses and nuclear factor (NF)-κB activation^13,18^, as well as secretion of IL-6, TNF, IL-17A, CXCL10/IP-10, CCL4, CCL20, and MCP-3 from anti-CD3 stimulated PBMC from T1D patients^18^. Because of GABA’s safety profile and ability to limit autoreactivity in preclinical models, there are ongoing clinical trials that administer GABA to individuals newly diagnosed with T1D (NCT02002130 and NCT03635437).

In regards to treating EAE, Steinman and colleagues showed that anti-seizure medications that increase GABAergic tone in the CNS (topiramate and vigabatrin) can inhibit EAE^7^. These drugs however primarily target other neurotransmitter systems (e.g., sodium and calcium channels) and enzymes^19,20^ and have adverse effects. Benzodiazepines and barbiturates are BBB-permeable GABA_A_-R positive allosteric modulators that can potentiate the opening of GABA_A_-R Cl^-^ channels, but only after a GABA_A_-R agonist opens the channel, and these drugs can be addictive. Accordingly, there is a need for safe BBB-permeable GABA_A_-R-specific agonists that could provide a new class of drugs for limiting the spreading of T cell autoreactivities in the CNS.

Homotaurine is an amino acid found in seaweed and it was identified as a compound that could interfere with the ability of amyloid peptide to form fibrils *in vitro*^21,22^. Subsequent studies found that oral homotaurine can pass through the BBB and limit amyloid plaque deposition in the brain of transgenic mice that over-expressed human amyloid protein^21,22^. Based on these observations, homotaurine (also known as Tramiprosate or Alzhemed™) was tested in a large double-blind phase III clinical trial for its ability to slow cognitive loss over 1.5 years in patients with Alzheimer’s disease^23–25^. While homotaurine treatment did not slow cognitive decline, it had an excellent safety profile. Recently, it has become appreciated that homotaurine can also act as a GABA_A_-R-specific agonist. Homotaurine has better pharmacokinetics than GABA (as discussed in^26,27^). We previously showed that oral homotaurine treatment inhibited both monophasic and RR-EAE (in the C57BL/6 MOG_35–55_ and SJL PLP_139-151_ mouse models, respectively) when treatment was initiated just after the appearance of clinical symptoms^26^. Histological analysis of their brains and spinal cords revealed reduced mononuclear cell infiltration and areas of myelin loss in the cerebellum and spinal cords of homotaurine-treated mice. Mechanistically, we observed that homotaurine significantly reduces the frequency of splenic PLP_139-151-_reactive IL-17A^+^ Th17 cells, as well as the frequency of splenic IFNγ (Th1) responses to PLP_139-151_, but increased IL-10-secreting responses, to PLP_139-151_ and the frequency of CD4^+^ and CD8^+^ Tregs^26^.

To further assess the therapeutic potential of targeting GABA_A_-Rs as a new avenue to help treat MS, we compared the therapeutic efficacy of GABA and homotaurine treatment on murine EAE, determined whether homotaurine treatment was efficacious when administered at an advanced stage of RR-EAE, assessed whether homotaurine administration could limit the spreading of T cell autoreactivity from the immunogen PLP_139-151_ to PLP_178-191_ within the CNS which is essential for disease progression^28,29^, and tested whether homotaurine treatment modulated the antigen-presenting activity of APCs. The results suggest that homotaurine is a promising candidate to help treat MS.

## Results

### Homotaurine, but not GABA, limits the development of EAE

The spreading of T cell autoreactivity to new myelin epitopes within the CNS is thought to be a major factor driving relapses in EAE and MS^28,30–32^. The inability of oral GABA to enter the CNS may make it ill-suited to limit the spreading of autoreactivities within the CNS. However, administered GABA may modulate cells of CNS-draining lymph nodes (which are outside of the BBB) as well as circulating activated/memory myelin-reactive T cells, and thereby impact the course of the disease. Hence, we tested the therapeutic efficacy of oral GABA versus homotaurine.

**Figure 1:**
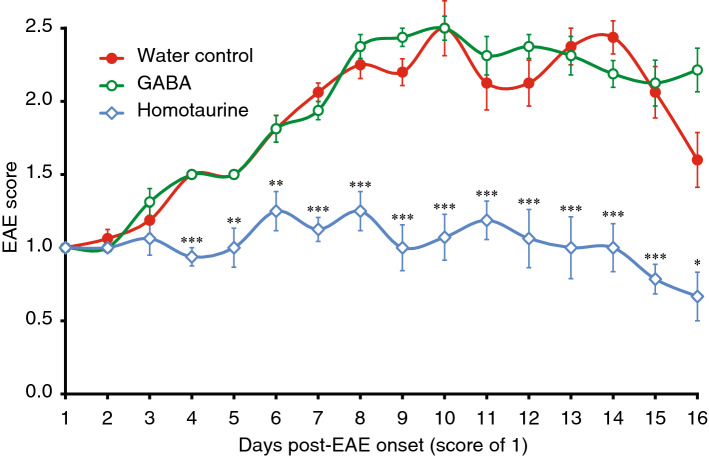
Homotaurine, but not GABA, limits the progression of EAE. C57BL/6 mice were immunized with MOG_35-55_ and monitored daily for clinical signs of EAE as described in “Methods”. Eleven to thirteen days post-immunization all of the mice developed EAE. When the mice reached an EAE score of 1 they were randomized to receive plain, water containing GABA (6 mg/ml) or homotaurine (0.25 mg/ml) for the rest of the 16-day observation period. Graph shows mean EAE scores ± SEM for mice that received plain water (red solid circles), GABA (green open circles), and homotaurine (blue open diamonds) after EAE onset. N = 8 mice/group. *p < 0.05, **p < 0.01, ***p < 0.001 vs. plain water control group by Student’s t-test.

C57BL/6 mice received MOG_33-55_ (as described in^26^) and all mice developed clinical signs of EAE between days 11–13 post-immunization. As individual mice reached an EAE score of 1 they were randomized to receive plain water or water containing GABA (6 mgs/ml, an effective dose in past studies of autoimmune disease^4,6,33^) or homotaurine (0.25 mg/ml) for the subsequent 16-day observation period. The disease course in the mice that received GABA was essentially the same as that in mice that received plain water (Fig. 1). In contrast, in homotaurine-treated mice, the disease severity remained near the initial trial entry score of 1 for about two weeks and then declined. The contrasting outcomes between GABA and homotaurine treatment suggest that homotaurine’s ability to pass through the BBB underlies its therapeutic effect.

### Homotaurine treatment initiated just before the second attack prevents EAE relapse

**Figure 2:**
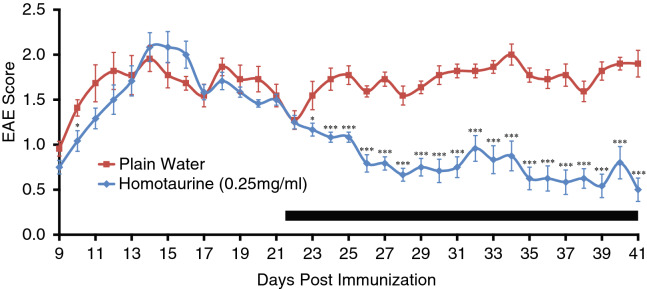
Homotaurine treatment initiated just before the second attack prevents EAE relapse. EAE was induced in SJL mice using PLP_139-151_ (as in^26^). Twenty-two days post-immunization, some mice were given homotaurine (0.25 mg/ml) continuously through their drinking water. The black bar indicates the period of homotaurine treatment. *p < 0.05, ***p < 0.001 vs. plain water control group by Student’s t-test. N = 11–12 mice/group**.**

We previously reported that PLP_139-151_ immunized SJL mice that were given homotaurine treatment at the first clinical signs of the disease (EAE score of 1) exhibited a reduced mean EAE score throughout the observation period compared to those given plain water and eventually displayed almost complete remission^26^. To more stringently test the robustness of this therapeutic approach we tested homotaurine’s ability to ameliorate disease when given at an advanced stage of EAE. SJL mice were immunized with PLP_139-151_ (as in^26^) and homotaurine treatment (0.25 mg/ml), was initiated 22 days later, just before the start of the expected relapse. Homotaurine treatment progressively reduced the severity of illness (Fig. 2).

### Homotaurine treatment limits the spreading of T cell autoreactivity within the CNS

In the SJL PLP_139-151_ disease model that we studied, the spreading of T cell autoimmunity from PLP_139-151_ to PLP_178-191_ is required for disease progression^28,29^ and has been shown to occur within the CNS and not the draining cervical lymph node or spleen^30,32^. T cell reactivity to PLP_178-191_ is observed to arise in the CNS during the secondary paralysis about 42 days post-PLP_139-151_ immunization^30^. Our observation that homotaurine, but not GABA, could modulate disease in mice with EAE suggests that homotaurine’s ability to pass through the BBB is key to its mode of action. To further test this hypothesis, we examined whether beginning homotaurine treatment during the first peak of clinical symptoms could modulate the spreading of T autoreactivity to a new myelin T cell epitope within the CNS.

**Figure 3:**
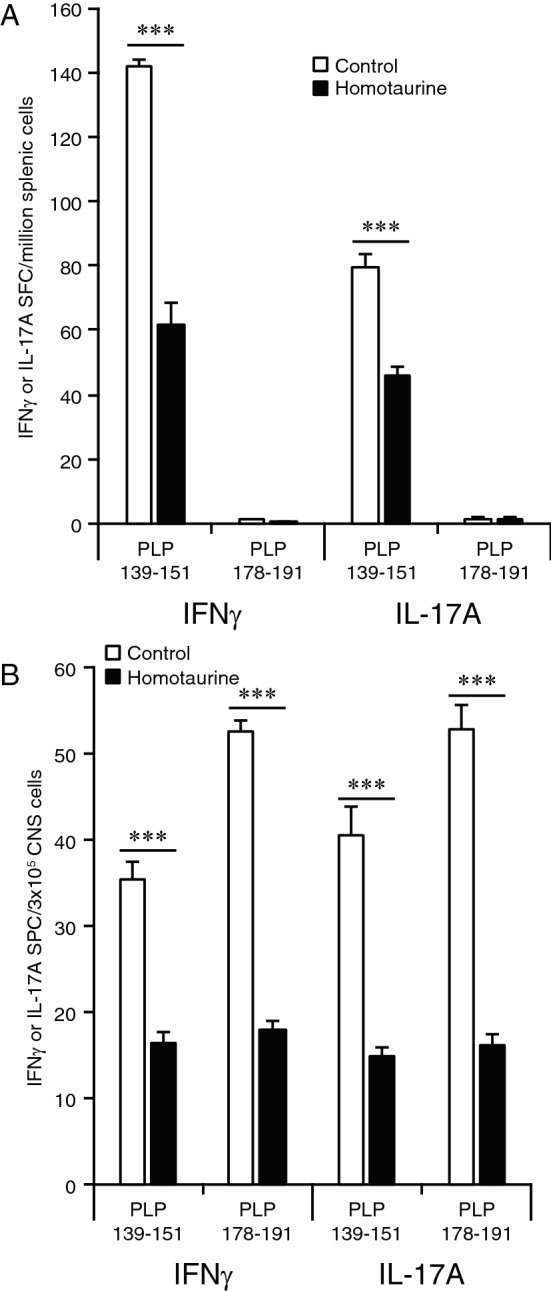
Homotaurine treatment limits the spreading of T cell autoreactivity within the CNS. SJL mice were immunized with PLP_139-151_ and 15 days later, near the first peak of clinical symptoms, mice were placed on plain water or containing homotaurine (0.25 mg/ml) for the rest of the experiment. Thirty days later, we analyzed the frequency of T cells which secreted IFNγ or IL-17A in response to PLP_139-151_ and PLP_178-191_ in the spleens (panel A) or CNS (panel B) of individual mice by ELISPOT. N = 6 mice/group. ***p < 0.001 by Student’s t-test.

We immunized SJL mice with PLP_139-151_ and 15 days later, a time point corresponding to the peak of PLP_139-151_-reactive T cells in the CNS^30^ and the first peak of clinical symptoms, we placed the mice on plain water or water containing homotaurine. Thirty days later, we analyzed the frequency of effector T cells which secreted IFNγ or IL-17A in response to PLP_139-151_ and PLP_178-191_ in the CNS and spleens of individual mice by ELISPOT (Fig. 3). In the spleen, we detected only PLP_139-151_-specific IFNγ- and IL-17A-secreting T cells, but not PLP_178-191_-responding T cells in both groups (Fig. 3A), consistent with past observations that there is no detectable spreading of T cell autoimmunity to PLP_178-191_ within the spleen^30^. In contrast, in the CNS, frequent IFNγ and IL-17A-secreting T cells were detected to both the priming immunogen PLP_139-151_ and to PLP_178-191_ in control mice given plain water. Thus, in the CNS, Th17 and Th1 autoimmunity spread from PLP_139-151_ to PLP_178-191_ as previously observed^30,32^. Importantly, in homotaurine treated mice, Th17 and Th1 responses were reduced to both PLP_139-151_ and to PLP_178-191_ relative to that found in plain water treated mice (Fig. 3B). Thus, homotaurine treatment limited the spreading of Th17 and Th1 responses from the priming immunogen to a new myelin epitope within the CNS. These observations, together with the reduced EAE clinical scores, support the notion that GABA_A_-R agonists with the ability to pass through the BBB can limit the spreading of pathogenic Th17 and Th1 responses in the CNS and inhibit disease progression.

### Homotaurine impairs the antigen presenting function of APCs to induce PLP_139-151_-specific T cell proliferation

**Figure 4:**
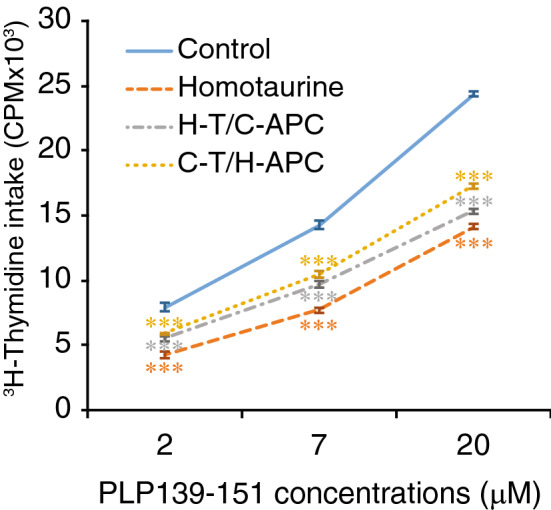
Homotaurine impairs the functions of splenic APCs. SJL mice were immunized with PLP_139-151_ and treated with, or without, homotaurine (0.25 mg/ml). Nine days later, CD4^+^ T cells were purified from their popliteal lymph nodes and T cell-depleted splenic mononuclear cells (APCs) were prepared by negative selection using microbeads and magnetic sorting. The T cells were mixed with APCs in a ratio of 5:1 and tested for T cell proliferation to the indicated concentrations of PLP_139-151_ for 3 days by ^3^H-thymidine incorporation. “Control” (solid blue line) represents ^3^H-thymidine incorporation by T cells and APCs isolated from untreated mice; “Homotaurine” (orange dashed line) shows ^3^H-thymidine incorporation by T cells and APCs isolated from homotaurine-treated mice**;** “H-T/C-APC” (grey dash/dotted line): T cells were isolated from homotaurine-treated mice and mixed with APCs from control untreated mice. “C-T/H-APC” (yellow dotted line): T cells were isolated from control mice and mixed with APCs from homotaurine-treated mice. Data are expressed as the mean values of each group ± SEM. The control cells without peptide stimulation had a CPM of 600–800 and the intra-group variation was less than 12%. N = six mice per group tested in two separate experiments. ***p < 0.001 vs. control by Student’s t-test.

We next examined whether homotaurine could modulate the ability of APCs to promote autoantigen-specific T cell mitogenesis. We purified CD4^+^ T cells and APC from PLP_139-151_ immunized SJL mice that were/were not treated with homotaurine for nine days. We co-cultured the CD4^+^ T cells from each group with APC isolated from plain water, or homotaurine-treated mice. We found that cultures of CD4^+^ T cells and APCs from the homotaurine-treated animals displayed the least levels of proliferation at all concentrations of PLP_139-151_ (Fig. 4). Co-cultures of T cells from the homotaurine-treated mice with APCs from the control mice also displayed reduced proliferation at all concentrations of peptide (“H-T/C-APC” in Fig. 4) consistent with the notion that homotaurine acts directly on T cells to limit their mitogenic potential. Notably, co-cultures of CD4^+^ T cells from the plain water-treated control animals with APCs from the homotaurine-treated animals exhibited a reduction in T cell proliferation (“C-T/H-APC” in Fig. 4, p < 0.001) at all peptide concentrations of peptide. Thus, homotaurine treatment inhibited T cell proliferation (as expected) but also limited the antigen-presenting functions of APCs*.*

## Discussion

Based on the success of GABA treatment to ameliorate T cell-mediated autoimmune diseases, we sought to extend this therapeutic approach to EAE and potentially MS. Our previous study showed that homotaurine treatment ameliorated both monophasic and RR-EAE when initiated just after the onset of symptoms. Here, we expanded those studies and also tested homotaurine treatment at advanced stages of RR-EAE since that is the most clinically relevant situation.

There are three major findings from the studies presented here. The first is that despite administering GABA at a level that effectively ameliorates T1D, RA, and coronavirus infection in mouse models, it had no effect on the disease course of EAE. In contrast, homotaurine treatment was an effective therapy. Since both GABA and homotaurine are GABA_A_-R agonists, we ascribe homotaurine’s therapeutic effectiveness to its ability to pass through the BBB. This contention is supported by our finding that homotaurine treatment limited the spreading of T cell autoreactivity from the priming immunogen PLP_139-151_ to PLP_178-191_ which has been shown to occur within the CNS and to be essential for driving relapses^28,30–32^. Since GABA also activates GABA_B_-Rs, it is possible that its lack of effect on EAE was due to GABA_B_-R mediated changes that counteracted the beneficial effects of GABA_A-_R activation. We do not prefer this scenario because GABA_B_-R agonists have been shown to (1) inhibit murine dendritic cell (DC) activation and immune cell chemotaxis^34,35^, (2) inhibit DC proinflammatory functions^34^, (3) alleviate collagen-induced arthritis and contact dermatitis in mouse models^34,35^, (4) delay T1D onset in NOD mice^36^, and (5) attenuate TLR4-induced inflammatory signaling in human PBMC^37^.

Interestingly, we also observed that homotaurine treatment significantly attenuated the activity of APCs in inducing antigen-specific T cell proliferation ex vivo. The attenuation of antigen-presenting activity by homotaurine could contribute to its inhibition of the spreading of T cell autoimmunity and disease progression. Although GABA can also inhibit the antigen-presenting activity of APCs, its failure to pass the BBB and inhibit EAE progression suggests that suppression of APC’s activity in the CNS may be necessary to inhibit the spreading of T cell autoimmunity and EAE progression in mice. Accordingly, these observations may shed new light on the immunoregulation of T cell autoimmunity within the CNS.

Second, our studies showed that homotaurine treatment beginning after the first wave of paralysis in an RR-EAE model greatly reduced the severity of the disease. This observation has potential clinical relevance since there is a critical need for late-stage therapeutic improvements for MS patients whose response to DMDs has diminished. Homotaurine had an excellent safety profile in long-term Alzheimer’s disease clinical trials making it an excellent candidate for clinical trials in MS patients and other neurological conditions in which limiting inflammation within the CNS is desirable.

Third, we observed that homotaurine treatment after the first wave of paralysis limited the spreading of Th17 and Th1 responses from PLP_139-151_ to PLP_178-191_ within the CNS. This is the first demonstration that a BBB-permeable GABA_A_-R agonist can limit the spreading of pathogenic Th17 and Th1 responses in the CNS. This diminution of epitope spreading is likely to be a major contributing factor to the reduced clinical severity of the disease in homotaurine-treated mice.

In addition, homotaurine may have modulated macrophages, DCs, microglia, and astrocytes in the CNS which express GABA_A_-Rs in ways that down-regulated their pro-inflammatory activities and contributed to the lower frequencies of autoreactive Th17 and Th1 cells in the CNS. Macrophages, DCs, microglia, and astrocytes also express GABA_A_-Rs and GABA treatment reduces their inflammatory activities^6–11^. Consistent with those observations, our co-culture studies showed splenic APC isolated from homotaurine-treated mice had reduced capacity to promote the proliferation of PLP_139-151_ -reactive T cell cells isolated from mice that did not receive homotaurine treatment. These data confirm and extend previous findings that GABA_A_-R agonist can modulate the functions of both T cells and APCs. However, further studies are needed to determine whether homotaurine treatment can modulate the activities of macrophages, DCs, microglia, and astrocytes in the CNS. Finally, glutamate excitotoxicity is a key feature of MS and EAE^38–41^ and homotaurine-mediated activation of CNS GABA_A_-Rs may have helped quell excitotoxicity.

Since GABA is at very low levels in tissues outside the CNS, we believe that the reason that immune cells express GABA-Rs is so that locally produced GABA can limit inflammation in the CNS. In this case, the activation of immune cell GABA-Rs is a natural mechanism to limit inflammation in the CNS, contributing to immune privilege in the CNS. This natural regulatory mechanism is insufficient to control the robust autoimmunity induced by priming with an encephalitic antigen, however, exogenous administration of homotaurine can inhibit the disease process. Similarly, although islet ß-cells secrete GABA, it is insufficient to prevent T1D in NOD mice, but exogenous GABA or homotaurine administration can prevent and/or reverse the disease^2–4,27^. Consistent with our observations, the administration of the benzodiazepine diazepam has been shown to inhibit EAE (e.g.,^42,43^). However, many benzodiazepines such as diazepam also bind to the mitochondrial translocator protein (TSPO, previously referred to as a “peripheral benzodiazepine receptor”), making it difficult to discern effects mediated through TSPO versus those through GABA_A_-Rs. Homotaurine’s safety record and its unique mechanisms of action make it an excellent candidate to test as an adjunct therapy with current DMDs for MS to potentially achieve improved therapeutic outcomes.

## Methods

### EAE induction

All experimental protocols were approved by the UCLA Animal Protection Committee and carried out in compliance with the ARRIVE guidelines and all relevant guidelines and regulations were followed for the experiment. Nine weeks old female C57BL/6 or SJL mice were obtained from the Jackson Laboratory and housed in a specific pathogen-free facility with free access to food and water. C57BL/6 mice were immunized subcutaneously with MOG_35-55_ (200 µg, > 95% purity, GenScript)) in 50% IFA containing Mycobacterium tuberculosis H37R (5 mg/ml, Difco) in multiple sites near the base of their tail on day 1 and injected intraperitoneally with pertussis toxin (200 ng/mouse) on day 0 and 2. Individual SJL mice were immunized with PLP_139-151_ (100 µg/mouse, > 95% purity, GenScript) using a similar protocol to that described for C57BL/6 mice. The mice were monitored for EAE onset daily: 0, no disease; 1, limp tail; 2, hind limb weakness; 3, complete hind limb paralysis; 4, quadriplegia; and 5, death. Mice that were in between the clear-cut gradations were scored intermediate in increments of 0.5. When the mice developed EAE with a score of 1 at 10–13 days post-immunization, they were randomized to receive plain water or water containing homotaurine (0.25 mg/ml) or GABA (6 mg/ml).

### Proliferation assays

Female SJL mice at 9 weeks of age were immunized with 100 µg/mouse of PLP_139-151_ peptide in 50% CFA in their foot-pads and treated with, or without, homotaurine in their drinking water (0.25 mg/ml). Nine days later, CD4^+^ T cells were purified from their popliteal lymph nodes (the responding T cells) and T cell-depleted splenic mononuclear cells (APCs) were prepared by negative selection using microbeads and magnetic sorting (BD PharMigen). The responding T cells were mixed with APCs in a ratio of 5:1, and were stimulated in triplicate with the indicated concentrations of PLP_139-151_ in 1% fetal calf serum HL-1 medium for 3 days. The mixed cells without peptide stimulation served as the negative control. During the last 16-h incubation, individual wells of cells were treated with 1 µCi ^3^H-thymidine and the T cell proliferation was determined by a β-counter.

### Statistics

EAE scores were evaluated using Kruskal–Wallis test. Pairwise comparisons were performed by 2-tailed Student’s t test. A P-value of < 0.05 was considered statistically significant.
